# Ki67/SATB1 ratio is an independent prognostic factor of overall survival in patients with early hormone receptor-positive invasive ductal breast carcinoma

**DOI:** 10.18632/oncotarget.5838

**Published:** 2015-10-15

**Authors:** Arvydas Laurinavicius, Andrew R. Green, Aida Laurinaviciene, Giedre Smailyte, Valerijus Ostapenko, Raimundas Meskauskas, Ian O. Ellis

**Affiliations:** ^1^ Faculty of Medicine, Vilnius University, Vilnius, Lithuania; ^2^ National Center of Pathology, Vilnius University Hospital Santariskiu Clinics, Vilnius, Lithuania; ^3^ Division of Cancer and Stem Cells, School of Medicine and Nottingham University Hospitals NHS Trust, University of Nottingham, Nottingham, United Kingdom; ^4^ National Cancer Institute, Vilnius, Lithuania

**Keywords:** immunohistochemistry, digital image analysis, breast cancer, HIF-1α, SATB1

## Abstract

Biological diversity of breast cancer presents challenges for personalized therapy and necessitates multiparametric approaches to understand and manage the disease. Multiple protein biomarkers tested by immunohistochemistry (IHC), followed by digital image analysis and multivariate statistics of the data, have been shown to be effective in exploring latent profiles of tumor tissue immunophenotype. In this study, based on tissue microarrays of 107 patients with hormone receptor (HR) positive invasive ductal breast carcinoma, we investigated the prognostic value of the integrated immunophenotype to predict overall survival (OS) of the patients. A set of 10 IHC markers (ER, PR, HER2, Ki67, AR, BCL2, HIF-1α, SATB1, p53, and p16) was used. The main factor of the variance was characterized by opposite loadings of ER/PR/AR/BCL2 and Ki67/HIF-1α; it was associated with histological grade but did not predict OS. The second factor was driven by SATB1 expression along with moderate positive HIF-1α and weak negative Ki67 loadings. Importantly, this factor did not correlate with any clinicopathologic parameters, but was an independent predictor of better OS. Ki67 and SATB1 did not reach statistical significance as single predictors; however, high Ki67/SATB1 ratio was an independent predictor of worse OS. In addition, our data indicate potential double prognostic meaning of HIF-1α expression in breast cancer and necessitate focused studies, taking into account the immunophenotype interactions and tissue heterogeneity aspects.

## INTRODUCTION

Remarkable progress in cancer research generates massive knowledge of a myriad of phenotypic complexities which can be conceptualized as manifestations of a small set of underlying organizing principles, representing biological hallmarks of cancer [1]. Similarly, molecular studies of breast cancer have uncovered significant biological diversity of the disease and lead to the concept of intrinsic biological subtypes, resulting in consensus therapy recommendations [2]. However, despite promising perspectives for personalized therapy, breast cancer management seems to remain caught between two worlds: the old world of familiar groupings defined by estrogen receptor (ER) and human epidermal growth factor receptor 2 (HER2) status and a new world of seemingly endless and complex ways to classify breast cancers [3].

Major effort is needed to fill the gap by translating this knowledge into practical therapeutic decision-making. Among the issues, recently addressed by the St Gallen-2015 [4], were the “semantic” classification of breast cancer subtypes by pathology-based biomarkers, biomarker prognostication dissecting the impact of the various gene signatures and pathologic variables in predicting the outcome of patients with early breast cancer as well as the challenges stemming from the intra- and inter-observer variability in the assessment of pathologic variables and the role of gene signatures.

“Multidimensional” complexity of breast cancer biology necessitates multiparametric measurement strategies to be implemented in clinical routine. This trend is represented by development of the multigene classifiers to complement traditional pathology methods, however, it remains to be seen whether more robust and simpler methods based on IHC could provide comparable information and be more suited to routine clinical practice [5]. Cuzick *et al* [6] have proposed the IHC4 score based on four IHC markers (ER, progesterone receptor (PR), HER2, and Ki67), commonly used in breast cancer, and suggested that the amount of prognostic information provided by the IHC4 was similar to that in the mRNA-based, 21-gene Genomic Health recurrence score. Subsequently, clinical utility of the IHC4 score supplemented by clinicopathologic parameters (IHC4+C score) [7] or by anti-apoptotic BAG1 protein measured by IHC [8] was reported. Yet, the IHC4-score could not outperform prognostic power of multigene expression tests [9, 10].

Combinatorial approach to IHC-based testing has been rather extensively explored for prognostic stratification of breast cancer patients [11], including the heterogeneity of the disease revealed by cluster analysis [12]. While it simulates the multivariate analysis approach used in multigene expression-based systems, the combined IHC biomarkers proposed are mostly based on visual qualitative or semi-quantitative evaluation. Lack of quantitative measurement methodologies resulting in poor reproducibility and low dynamic range of the data can be a major drawback of the IHC-based tissue protein testing.

Recent advances of high-resolution scanning of microscopic slides and digital image analysis (DIA) bring new levels of accuracy, reproducibility and capacity that can be achieved by IHC-based testing [13]. In addition to improved quantification and analytical power, DIA can utilize spatial aspects of IHC-based tests to uncover intra-tissue heterogeneity of the biomarker expression along with measurement of multiple biomarker in the tissue [14, 15].

We have previously demonstrated the feasibility to obtain multivariate IHC characteristics of breast tumor tissue, based on DIA of a set of 10 IHC markers (ER, PR, HER2, Ki67, androgen receptor (AR), BCL2, HIF-1α, SATB1, p53, and p16) on tissue microarrays (TMA) [16]. Factor analysis of the data proved to be an efficient exploratory tool clarifying latent interdependencies in the IHC profiles. In particular, we found that a major factor of the aggressive disease behavior, associated with histological grade and relevant intrinsic subtypes, was characterized by opposite loadings of ER/PR/AR/BCL2 and Ki67/HIF-1α. Remarkably, the second major factor of variation was represented by predominant SATB1 along with HIF-1α; however, this factor was not associated with any clinicopathologic parameters in this study. While biological and clinical meaning of this factor remained unclear, we hypothesized that HIF-1α and SATB1 co-expression may convey important biological messages other than the aggressiveness of the disease reflected by Ki67 expression and histological grade.

In the present study, we present multivariate analysis of IHC data in 107 patients with early HR-positive invasive ductal breast carcinoma and prognostic value of the tumor immunophenotype to predict overall survival (OS) of the patients. Our results highlight independent prognostic value of the immunophenotype driven by the SATB1 expression, in covariance with Ki67 and HIF-1α expression.

## RESULTS

### Patient and tumor characteristics

Patient and tumor characteristics are presented in Table 1, including the data on adjuvant therapies available in 104 patients. Since the intrinsic subtypes were subdivided based on the visual evaluation of the IHC images, the DIA results on ER, PR, HER2, and Ki67 do not strictly correspond to the conventional cut-off values used for the definition of intrinsic subtypes [2]. Pairwise correlations between the IHC markers are presented in the Table 2.

**Table 1:** Patient and tumor characteristics

	Luminal A	Luminal B	Luminal B HER2+	p
Age group				n.s
Age ≤ 55 year (*n* = 47)	24 (40%)	14 (48%)	9 (50%)	
Age > 55 year (*n* = 60)	36 (60%)	15 (52%)	9 (50%)	
Histological grade				<0.0001
1	20 (33%)	3 (10%)	2 (11%)	
2	37 (62%)	8 (28%)	6 (33%)	
3	3 (5%)	18 (62%)	10 (56%)	
T				n.s.
1	37 (62%)	15 (52%)	9 (50%)	
2	23 (38%)	14 (48%)	9 (50%)	
N				n.s.
0	34 (57%)	14 (48%)	9 (50%)	
1	26 (43%)	15 (52%)	9 (50%)	
Endocrine therapy	56 (94%)	23 (82%)	13 (75%)	<0.005
Chemotherapy	31 (53%)	20 (71%)	12 (71%)	<0.007
Radiotherapy	52 (88%)	25 (89%)	12 (71%)	<0.05
Transtuzumab therapy	0 (0%)	0 (0%)	7 (41%)	<0.0001
% positive cells by immunohistochemistry measured by digital image analysis (mean ± SD)*				
ER	78 ± 15^a^	65 ± 30^b^	57 ± 26^b^	<0.0007
PR	53 ± 30^a^	41 ± 35^a^	25 ± 31^b^	<0.004
AR	48 ± 20^a^	38 ± 22^a^	29 ± 21^b^	<0.003
BCL2	55 ± 12^a^	49 ± 21^b^	32 ± 26^b^	<0.0002
HER2	9 ± 12^a^	7 ± 11^a^	38 ± 24^b^	<0.0003
Ki67	13 ± 7^a^	34 ± 14^b^	23 ± 14^c^	<0.0001
p53	18 ± 17	31 ± 30	21 ± 18	n.s.
p16	17 ± 8	17 ± 12	15 ± 9	n.s.
HIF-1α	9 ± 6	11 ± 10	13 ± 10	n.s.
SATB1	14 ± 10	14 ± 10	14 ± 8	n.s.

* Statistical significance of variation between the groups tested by one-way ANOVA (logarithm-transformed values of HER2, Ki67, p53, p16, HIF-1α, SATB1 were used for the analysis, however, original values are presented in the table).

** The labels ^a b c^ indicate pairwise comparisons with statistical significance at *p* < 0.05.

**Table 2:** Pairwise correlations between the immunohistochemical markers of ductal carcinoma of the breast

	ER	PR	AR	BCL2	HER2	Ki67	p53	p16	HIF-1α
PR	**0.23**								
AR	**0.46**	**0.46**							
BCL2	**0.44**	**0.29**	**0.31**						
HER2	− .13	**− .22**	− .18	**− .33**					
Ki67	**− .21**	**− .25**	**− .27**	**− .19**	− .04				
p53	− .02	0.13	− .06	− .12	**0.20**	0.01			
p16	− .04	0.10	0.17	0.08	0.08	**− .26**	0.12		
HIF-1α	**− .35**	**− .34**	**− .40**	**− .38**	0.01	**0.20**	− .17	− .06	
SATB1	**− .27**	− .03	− .15	**− .21**	0.11	− .14	**0.19**	0.11	**0.54**

Statistically significant (*p* < 0.05) correlation coefficients are presented in bold.

### Factor analysis of the tumor immunophenotype

**Figure 1:**
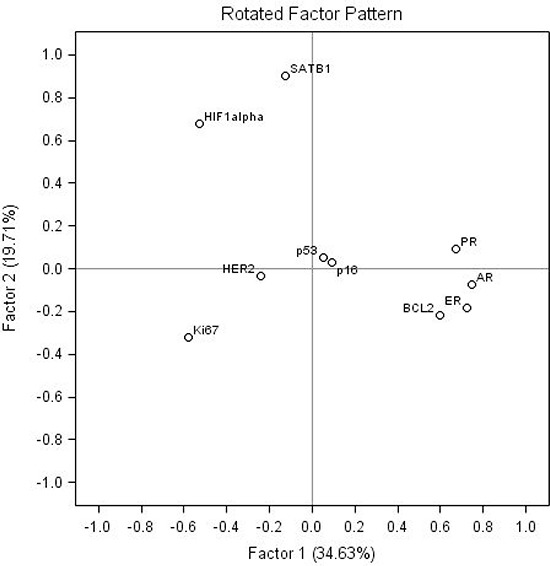
Rotated factor pattern: loadings of the factors 1 and 2 plotted

Factor analysis of the complete data set of the 10 IHC markers revealed essentially the same intrinsic factors as in the previous study [16]; rotated factor pattern is presented in the Table 3, the loadings of the factors 1 and 2 plotted in Figure 1. Altogether, the five factors explained 75% of the variance in the dataset.

**Table 3:** Rotated factor pattern of the immunophenotype variation

	Factor 1	Factor 2	Factor 3	Factor 4	Factor 5
ER	0.72603	−0.18378	−0.14225	0.10913	−0.27589
PR	0.67463	0.09000	0.34085	−0.26292	0.04576
AR	0.74839	−0.07607	0.12587	−0.06703	0.08694
BCL2	0.59629	−0.21926	−0.24701	−0.30652	0.10184
HER2	−0.24123	−0.03382	0.17975	0.86172	0.03656
Ki67	−0.58024	−0.31861	0.23088	−0.41239	−0.31604
p53	0.05071	0.05017	0.89504	0.15468	0.05463
p16	0.09120	0.02786	0.05840	0.04296	0.94506
HIF-1α	−0.52664	0.67899	−0.21750	−0.11842	−0.09514
SATB1	−0.12619	0.90298	0.17020	0.04825	0.08304

Factor 1 and 2 represented major portion of the variance explained by the five factors extracted (35 and 20%, respectively). Factor 1 was characterized by strong positive loadings of HR (ER, PR, AR) and BCL2 as well as strong negative loadings of Ki67 and HIF-1α. Factor 2 was characterized by strong positive loadings of SATB1 and HIF-1α (0.90 and 0.68, respectively) along with weak negative loading (−0.32) of Ki67. Of note, HIF-1α was also involved in factor 1 with the loading of −0.53. Factors 3, 4 and 5 altogether represented the remaining 45% of the variance explained by the five factors. The factors were characterized by positive loadings of single biomarkers: factor 3 (p53), factor 4 (HER2), and factor 5 (p16). The factor scores revealed normal distribution (not shown).

### Associations between the tumor immunophenotype and the conventional characteristics of the ductal carcinoma of the breast

**Figure 2:**
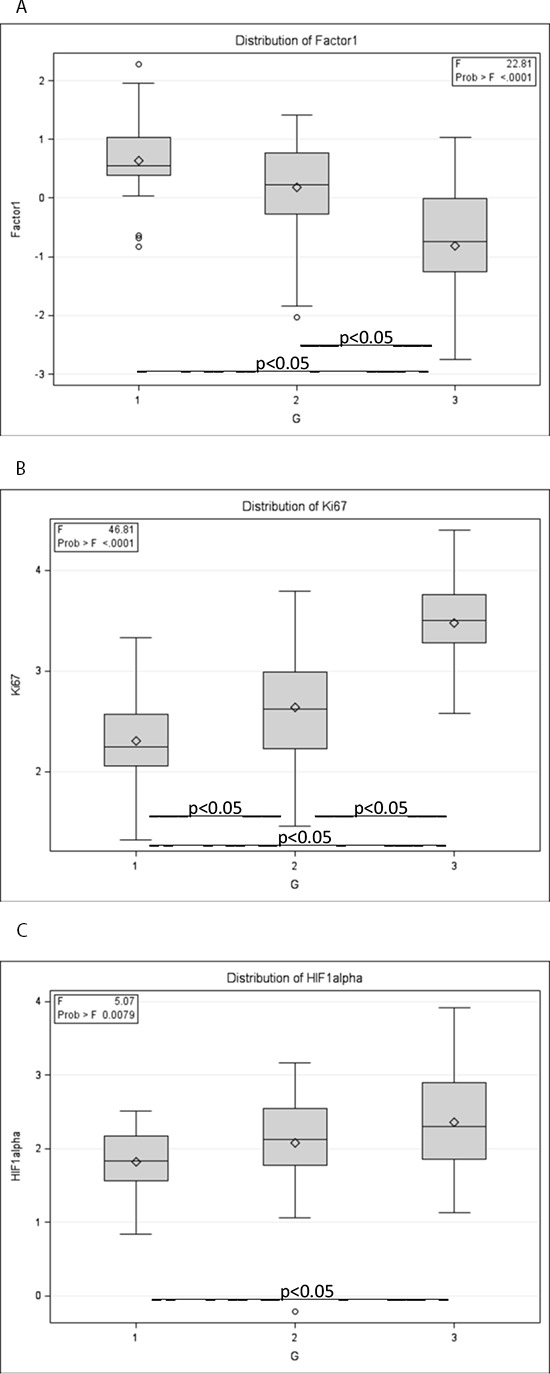
Associations of the tumour immunophenotype to histological grade (G) ANOVA box whisker plots of **A.** factor 1 scores; **B.** Ki67; **C.** HIF-1α.

In addition to the tumor immunophenotype associations to the intrinsic subtypes presented in the Table 1, we explored potential associations of the tumor immunophenotype the histological grade (G), tumor stage (T), node status (N), and patient's age group. The histological grade presented significant associations to the factor 1 and the corresponding IHC markers (Figure 2): higher grade presented with lower levels of factor 1 scores (*p* < 0.0001), ER (*p* < 0.05), PR (*p* < 0.03), AR (*p* < 0.0003), BCL2 (*p* < 0.05) and higher levels of Ki67 (*p* < 0.0001) and HIF-1α (*p* < 0.001) expression. Neither factor 2, 3, 4, 5 scores nor SATB1, p53, HER2, p16 expression revealed significant associations to the other tumor characteristics.

### Predictors of the overall survival of the patients

**Figure 3:**
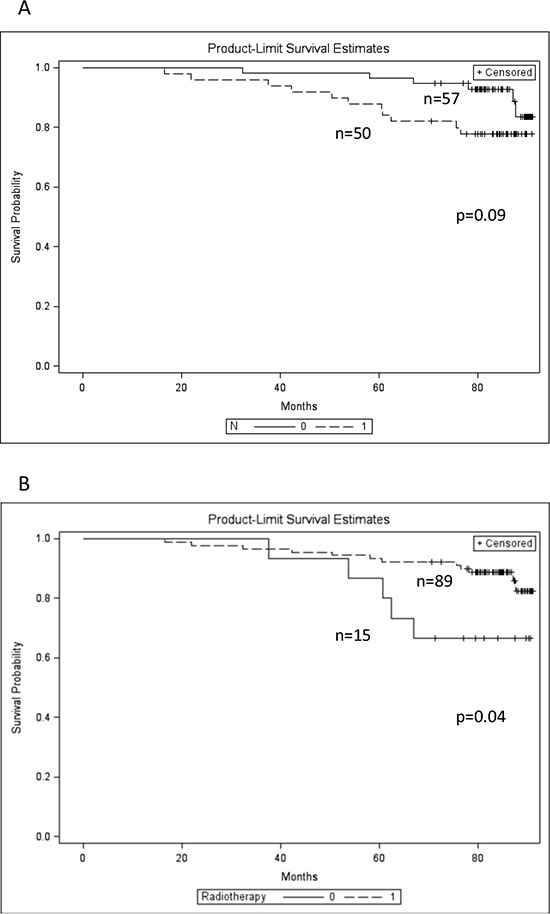
Kaplan-Meier overall survival plots of the patients with different lymph node status and radiotherapy applied **A.** lymph node status: N1 (dashed line), N0 (solid line); **B.** radiotherapy applied (dashed line), not applied (solid line).

Mean duration of follow-up after the surgery was 80.4 ± 13.9 months (range 17 to 91 months, median 84). Eighteen patients died during the follow-up period. The histological grade (G), tumor stage (T), node status (N), and patient's age group did not predict the OS by product-limit analysis. Radiotherapy was associated with better OS (*p* = 0.04), while the N1 status was associated with worse OS, however, not reaching the level of significance (*p* = 0.09), Figure 3A and 3B.

Multiple variable models were developed to account simultaneously for the conventional and immunophenotype characteristics of the tumors (Table 4). Model#1 (*p* = 0.034) was derived from a dataset consisting of patient's age group, T, N, G and the tumor immunophenotype represented by the factor scores and revealed better OS predicted by the factor 2 scores (hazard ratio 0.541, *p* = 0.047). Model#2 (*p* = 0.012) was achieved by adding to the dataset a product of the factor 1 and 2 scores thus testing a hypothesis that a ratio of factor 2 and inverted factor 1 scores are additive on OS prediction (hazard ratio 0.492, *p* = 046). Model#3 (*p* = 0.007) was derived from a dataset represented by only primary IHC variables as well as their ratios; it revealed worse OS predicted by high Ki67/SATB1 (hazard ratio 2.028, *p* < 0.007). Model#4 (*p* = 0.017) was derived from a dataset consisting of patient's age group, T, N, G and the tumor immunophenotype represented by the primary IHC variables as well as their ratios; it revealed worse OS predicted by the N1 and high Ki67/SATB1 (hazard ratio 2.883 (*p* < 0.049) and 1.778 (*p* < 0.006), respectively).

**Table 4:** Cox multivariate regression models to predict overall survival

	Hazard ratio	95% confidence limits	*P* value
Model#1			0.0339
Factor 2 score	0.541	(0.295, 0.992)	0.0470
Model#2			0.0120
Factor 2 score * Factor 1 score	0.492	(0.245, 0.988)	0.0461
Model#3			0.0235
Ki67/SATB1 ratio	2.028	(1.037, 3.965)	0.0068
Model#4			0.0168
N1	2.883	(1.004, 8.274)	0.0491
Ki67/SATB1 ratio	1.778	(1.183, 2.671)	0.0056

**Figure 4:**
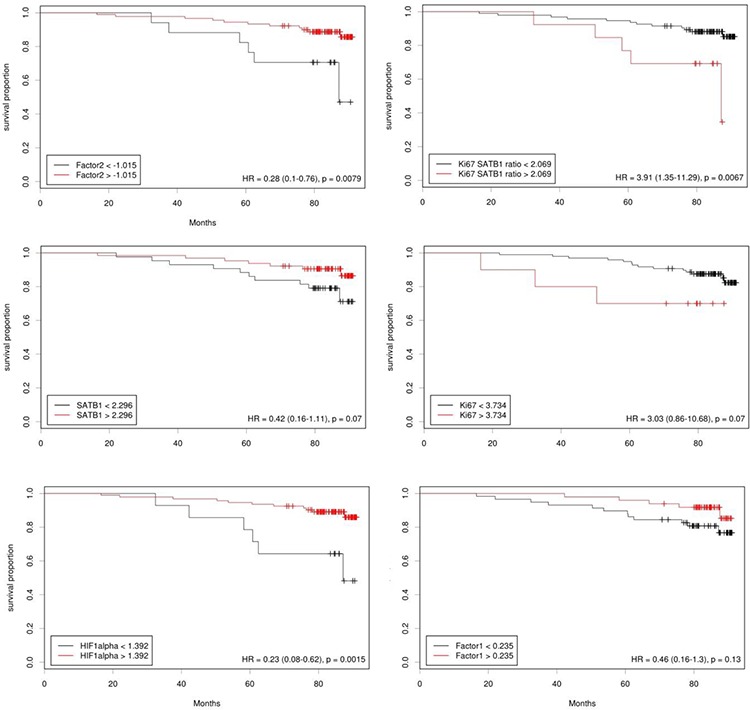
Cutoff values for the predictors of overall survival

In addition, relevant IHC markers and tumor immunophenotype factor scores were dichotomised using the web-based tool “Cutoff Finder” [17] and were analyzed using Kaplan–Meier estimates and log rank tests. While no significant cutoff values could be established for Ki67, SATB1, and factor 1 scores, the factor 2 scores (*p* = 0.008) and Ki67/SATB1 ratio (*p* = 0.0067) allowed dichotomization of patients into prognostic groups (Figure 4). Interestingly, HIF-1α, as a single predictor, allowed dichotomization (*p* = 0.002) of the patients regarding their OS, although this marker did not reveal independent prognostic value in the multivariate Cox regression models.

## DISCUSSION

Our study provides evidence that combined tumor tissue IHC-based prognostic biomarkers can be derived by the multivariate analysis of IHC DIA data. In the cohort of 107 patients with HR-positive ductal carcinoma of the breast, we detected latent interdependencies of 10 IHC biomarkers and tested their value to predict OS of the patients. Remarkably, our study reveals that OS could be better predicted by the integrated factor score driven by SATB1 along with HIF-1α expression rather than the immunophenotype represented by HR and BCL2 covariance inversely related to Ki67 positivity, also correlated to the histological grade of the tumors. Furthermore, the extracted immunophenotypic pattern directed to the discovery of a significant prognostic model represented by the Ki67/SATB1 ratio, while these markers did not reach statistical significance as single predictors.

The design of our study enabled us to avoid significant human evaluation bias while obtaining the results: we performed an automated DIA of TMA stained for 10 IHC markers, obtaining continuous tumor tissue immunophenotype data represented by the percentage of positive cells. As in our previous study [16], factor analysis revealed orthogonally independent latent factors governing the variance of the immunophenotype; importantly, the factor pattern remained essentially the same in the extended HR-positive patient cohort, now supplemented with accumulated OS data.

We found that the major factor of the tumor immunophenotype variation was characterized by a strong inverse relation between the expression of ER, PR, AR along with anti-apoptotic marker BCL2, on one side, and Ki67 and HIF-1α, on the other side. The factor 1 scores as well as Ki67 and HIF-1α values were associated with histological grade (Figure 2), therefore, could be interpreted as an expression of aggressiveness of the disease. Nevertheless, neither the factor 1 score nor Ki67 expression, as a single variable, reached significant impact on the OS.

The factor 2 scores, represented mainly by SATB1 and HIF-1α co-expression did not correlate to any clinical or pathology variables in the study. Surprisingly, the factor 2 scores were significant predictors of better OS in the univariate and multivariate models. Also, HIF-1α (*p* = 0.0015) and SATB1 (not significant, *p* = 0.07) revealed positive effects on OS in univariate models. Furthermore, the combination of factor 1 and factor 2 scores, represented by their product (or, factor 2 divided by reciprocal factor 1) slightly improved the predictive power of better OS and exceeded that of the factor 2 and the multivariate Cox regression model. Accordingly, the high Ki67/SATB1 ratio, alone or together with the lymph node involvement (N1), predicted worse OS. In other words, it can be interpreted that high tumor proliferative rate, measured by Ki67 expression, in the context of low SATB1 (and relatively low HIF-1α) expression was an independent predictor of worse OS. Similarly, SATB1 predicted better OS when co-expressed with HIF-1α.

Our study sheds light on SATB1 – a relatively new and less-explored biomarker in breast cancer. SATB1 is a genome organizer that recruits chromatin-remodeling enzymes to regulate chromatin structure and gene expression, while its role as a prognostic factor and a potential target for therapy remains controversial. It has been implicated to promote growth and metastasis of breast cancer and indicate poor prognosis [18, 19], however, this has not been confirmed by other studies [20–23]. In particular, the expression levels of SATB1 mRNA in 2058 breast cancer samples were not related to disease-free survival among ER negative cancers, however, high SATB1 expression among ER positive tumors showed beneficial effect on prognosis; nevertheless, even in ER positive cancer no independent prognostic value in multivariate analysis with standard parameters was observed [21]. More recent studies are suggestive of adverse prognostic value for SATB1: significantly improved overall survival has been shown for homozygous SATB1 − 3600T/− 3363A/− 2984C haplotype carriers with expected lower SATB1 promoter activity in a cohort of breast cancer patients [24]. Kobierzycki *et al* [25] found a moderate positive correlation between Ki67 and SATB1 expression in ER-negative patients (*r* = 0.392, *p* = 0.032) but not ER-positive breast cancer, which may indicate an indirect role of SATB1 in the cancer cell proliferation. Liu et al [26] reported on the relationships between SATB1, HER2, HR expression and clinicopathologic characteristics in breast cancer tissues: SATB1, HER2 and SATB1/HER2 co-expression correlated with higher histological grade and were independent risk factors of worse survival. The role of SATB1 remains controversial and appears to be specific to the type of cancer, in particular, high levels of SATB1 expression are associated with poor prognosis in colorectal cancer [27], loss of SATB1 – with poor prognosis in lung squamous cell carcinoma [28], expression of SATB1 was an independent predictor of a significantly shorter recurrence-free survival and OS in pancreatobiliary type, but not in intestinal type adenocarcinomas of pancreas; moreover, SATB1 expression predicted an improved response to adjuvant chemotherapy in both tumor types [29].

In the present study of HR-positive breast cancer, we did not find significant associations of SATB1 IHC expression to the histological grade, T or N stage, but demonstrated positive association with HIF-1α expression by pairwise correlation (*r* = 0.54, *p* < 0.05) and factor analysis. Weak inverse correlation between SATB1 and ER (*r* = −0.27, *p* < 0.05) noted in our study may indeed suggest of possible differences of SATB1 role in HR-positive and HR-negative breast cancer. Importantly, our data supports the evidence that SATB1 is associated with better OS in HR-positive breast cancer (although did not reach statistical significance as a single predictor, *p* = 0.07), especially, when co-expressed with and HIF-1α (factor 2). Furthermore, high ratio of Ki67/SATB1, representing the interaction of factors 1 and 2 in our study, served as the best independent predictor of worse OS in our dataset.

Multivariate analysis of the cancer immunophenotype uncovered intriguing latent interactions between the biomarkers studied and could explain some controversies in their prognostic significance. Ki67 and HIF-1α are commonly viewed as the markers of poor prognosis in breast cancer. According to our data, Ki67 and HIF-1α were both associated with higher histological grade and lower expression of HR and BCL-2; this finding can be interpreted as a reflection of the well-known association between the proliferative activity and low differentiation of the tumors. However, Ki67 and HIF-1α expression is also influenced by another, independent factor, best highlighted by the expression of SATB1. While pathophysiology of this second factor remains to be uncovered, it becomes apparent that in the context of SATB1 expression, Ki67 and HIF-1α reveal inverse covariance. Therefore, Ki67 and HIF-1α expression can carry at least duplicate pathophysiological messages to be considered in prognostic modelling of the disease. In particular, our data can be interpreted in a way that high Ki67 expression in the context of low SATB1 and low HIF-1α (expressed by low Factor 2 scores or low SATB1/Ki67 ratio) was a better predictor of poor OS, rather than high Ki67 in the context of low HR expression and high histological grade.

The “double” effect of HIF-1α expression in our study could be seen as a paradox: while it correlated with Ki67 expression (Factor 1) and the histological grade, it also correlated with SATB1 expression (Factor 2) and better OS as a single but not independent predictor. HIF-1α is broadly expressed in many human cancers and frequently correlates with poor prognosis; it affects many key aspects of tumor aggressiveness and represents an attractive target for anti-cancer therapies [30]. In breast cancer, HIF-1α is associated with high histological grade, lymph node metastasis, large tumor size, high proliferation rate, negativity of HR, HER2 positivity as well as shorter disease-free and OS [31–34]. However, interpretation of the beneficial prognostic effect of HIF-1α along with SATB1 co-expression is less straightforward. The interactions between the two markers have not been investigated. One likely explanation of this phenomenon is consistent with the observation that different regulation pathways of HIF-1α overexpression exist in breast cancer: (1) hypoxia induced, perinecrotic HIF-1α overexpression with strong expression of hypoxia associated genes, which is associated with a poor prognosis; and (2) diffuse HIF-1α overexpression lacking major hypoxia associated downstream effects, resulting in a more favorable prognosis [35, 36]. Since TMA were randomly sampled from the tumor tissue in our study, they are expected to represent diffuse rather than localized expression of the markers.

Our present study contains some limitations. Firstly, it is based on a relatively small patient cohort, which could be insufficient to achieve statistical power with regard to prognostic value of well-established clinico-pathological parameters and modes of therapy. Nevertheless, the dataset was sufficient to reveal the prognostic value of the quantified IHC biomarkers in the single and multivariate models. Importantly, our cohort also revealed association of radiotherapy with better OS as in other observational studies [37, 38]; however, neither radiotherapy nor the other therapy modes reached statistical significance as independent OS predictors. Also, lymph node involvement (N) became significant in the context of IHC data in the multivariate Cox regression. Secondly, we obtained the tumor immunophenotype data from single IHC tests performed on consecutive TMA sections. While this approach uncovered novel immunophenotype interactions with potential clinical significance, further studies on the biomarker co-expression and spatial distribution in the tumor tissue are required. In particular, focus on multiplex testing of SATB1, HIF-1α, Ki67, HER2 on the whole tissue sections could provide most direct answers to the puzzle.

In summary, our study confirms that factor analysis of multiple IHC biomarkers, measured by automated DIA, is an informative method to discover latent interdependencies in breast cancer tissue immunophenotype. In addition to the main factor, reflective of the aggressive disease, characterized by opposite loadings of ER/PR/AR/BCL2 and Ki67/HIF-1α, and associated with high histological grade, the second important factor behind the immunophenotype variance was driven by SATB1 expression along with moderate positive HIF-1α and weak negative Ki67 loadings. Importantly, this factor did not correlate with any clinicopathologic parameters in our study; however, it was an independent predictor of better OS. As single predictors of OS, Ki67 and SATB1 did not reach statistical significance; however, high Ki67/SATB1 ratio was an independent predictor of worse OS, most likely, representing the effect of SAT1-driven tumor immunophenotype. HIF-1α was significant as single but not as an independent predictor of better OS. Our data support a notion of potential double prognostic meaning of HIF-1α expression in breast cancer and necessitate focused studies taking into account the latent immunophenotype interactions and tissue heterogeneity aspects.

## MATERIALS AND METHODS

### Study population and clinical methods

Tumor samples were prospectively collected in our previous study [16] from 203 patients with an invasive ductal carcinoma of the breast treated at the National Cancer Institute (previously, Oncology Institute of Vilnius University) and investigated at the National Center of Pathology during the period of 2007 to 2009. Informed consent was obtained and documented in writing before study entry. The study was approved by the Lithuanian Bioethics Committee. Data on patient survival and adjuvant therapy modes was obtained from the National Cancer Institute.

IHC was performed on TMAs as previously described [16]. Briefly, 1 mm-diameter cores were punched from areas throughout the tumor randomly selected by a pathologist (4 cores per patient). Paraffin sections of the TMAs were cut 3 μm-thick. IHC was performed using Ultraview DAB detection kit on Ventana BenchMark XT staining system (Ventana Medical Systems, Tucson, Arizona, USA). Immunohistochemistry for ER, PR, HER2, AR, Ki67, p53, p16, BCL2, SATB1 and HIF-1α was performed using the SP1, 1E2 and 4B5 (Ventana), SP107 (Spring), MIB-1 (DAKO), DO-7 (Novocastra), E6H4 (CINtec), 124 (DAKO), EPR3895 and EP1215Y (Epitomics) antibodies, respectively. Digital images were captured using the Aperio ScanScope XT Slide Scanner (Aperio Technologies, Vista, CA, USA) under 20x objective magnification.

### Digital image analysis

The DIA was performed using Aperio Genie Classifier was trained to recognize tumor tissue, stroma and background (glass). The Genie classifier was combined with Aperio Membrane v9 and Aperio Nuclear v9 algorithms. The percentage of tumor cells with complete membranous (HER2 and BCL2) and positive nuclear (ER, PR, AR, Ki67, p53, p16, SATB1 and HIF-1α) staining was used for further analyses. The percentage of positive cells was calculated from positive and negative cells summed from each patient's TMA cores with a threshold of total number of tumor cells per patient set at >99. TMA cores containing ductal carcinoma *in situ* were excluded from evaluation. The IHC and DIA images are presented in the previous publication [16].

In our previous study consisting of 203 patients with ductal breast carcinoma, a total of 109 patients with a complete set of 10 IHC markers were available for multivariate analyses (after exclusion of the cases with non-informative TMA cores for at least of one of the 10 IHC markers), including 85 HR-positive cases. For the present study, we have produced an additional TMA to fill the data gaps and to achieve a full dataset in an additional 22 cases resulting in a total of 107 HR-positive cases representing Luminal A, Luminal B, and Luminal B HER2 positive tumors. HR-positivity was defined as ER and/or PR positivity in at least 1% of tumor cells, a cutoff of Ki67 ≥ 14% was used for Luminal B category, HER2 positivity defined based on IHC3+ or IHC2+ verified by HER2 FISH test.

### Statistical analysis

Summary statistics and distribution analyses were performed with significance tests based on one-way ANOVA. Since distributions of HER2, Ki67, HIF-1α, SATB1, p53, and p16 DIA results revealed a positive skew, logarithm-transformed values were used for parametric statistics. For the sake of readability, the prefix “log” is not used in the text or graphs when referring to these markers.

Factor analysis on a DIA data set of 10 IHC markers was performed as previously [16], using factoring method of principal component analysis. Five factors were retained; general orthomax rotation of the initial factors was performed.

Pearson's correlation was performed to test the pairwise linear relationships between the continuous variables as a preparatory step for factor analyses. Chi-square test and Fisher's exact test were used to estimate significant associations in non-parametric statistics. Product-limit estimates were used to summarize OS data and the log rank test was used for comparing OS distributions. OS was defined as the time from the breast surgery to the patient's death. Cox proportional hazards analysis was used to develop a multiple variable models to predict time to death. A combination of forward, backward, and stepwise procedures was used to arrive at the final model. Bonferroni's multiple comparison testing and corrections were applied where appropriate. Continuous variables were dichotomised to predict OS using the web-based tool “Cutoff Finder” [17]. Statistical significant set was at *p* < 0.05. Statistical analysis was performed with SAS 9.3 software.

## References

[1] Hanahan D, Weinberg RA (2011). Hallmarks of cancer: the next generation. Cell.

[2] Goldhirsch A, Wood WC, Coates AS, Gelber RD, Thurlimann B, Senn HJ (2011). Strategies for subtypes—dealing with the diversity of breast cancer: highlights of the St Gallen International Expert Consensus on the Primary Therapy of Early Breast Cancer. Ann Oncol.

[3] Prat A, Ellis MJ, Perou CM (2011). Practical implications of gene-expression-based assays for breast oncologists. Nat Rev Clin Oncol.

[4] Esposito A, Criscitiello C, Curigliano G (2015). Highlights from the 14(th) St Gallen International Breast Cancer Conference 2015 in Vienna: Dealing with classification, prognostication, and prediction refinement to personalize the treatment of patients with early breast cancer. Ecancermedicalscience.

[5] Rakha EA, Ellis IO (2011). Modern classification of breast cancer: should we stick with morphology or convert to molecular profile characteristics. Adv Anat Pathol.

[6] Cuzick J, Dowsett M, Pineda S, Wale C, Salter J, Quinn E, Zabaglo L, Mallon E, Green AR, Ellis IO, Howell A, Buzdar AU, Forbes JF (2011). Prognostic value of a combined estrogen receptor, progesterone receptor, ki-67, and human epidermal growth factor receptor 2 immunohistochemical score and comparison with the genomic health recurrence score in early breast cancer. J Clin Oncol.

[7] Barton S, Zabaglo L, A'Hern R, Turner N, Ferguson T, O'Neill S, Hills M, Smith I, Dowsett M (2012). Assessment of the contribution of the IHC4+C score to decision making in clinical practice in early breast cancer. Br J Cancer.

[8] Afentakis M, Dowsett M, Sestak I, Salter J, Howell T, Buzdar A, Forbes J, Cuzick J (2013). Immunohistochemical BAG1 expression improves the estimation of residual risk by IHC4 in postmenopausal patients treated with anastrazole or tamoxifen: a TransATAC study. Breast Cancer Res Treat.

[9] Sgroi DC, Sestak I, Cuzick J, Zhang Y, Schnabel CA, Schroeder B, Erlander MG, Dunbier A, Sidhu K, Lopez-Knowles E, Goss PE, Dowsett M (2013). Prediction of late distant recurrence in patients with oestrogen-receptor-positive breast cancer: a prospective comparison of the breast-cancer index (BCI) assay, 21-gene recurrence score, and IHC4 in the TransATAC study population. Lancet Oncol.

[10] Dowsett M, Sestak I, Lopez-Knowles E, Sidhu K, Dunbier AK, Cowens JW, Ferree S, Storhoff J, Schaper C, Cuzick J (2013). Comparison of PAM50 Risk of Recurrence Score With Oncotype DX and IHC4 for Predicting Risk of Distant Recurrence After Endocrine Therapy. J Clin Oncol.

[11] Rakha EA, Reis-Filho JS, Ellis IO (2010). Combinatorial biomarker expression in breast cancer. Breast Cancer Res Treat.

[12] Soria D, Garibaldi JM, Ambrogi F, Green AR, Powe D, Rakha E, Macmillan RD, Blamey RW, Ball G, Lisboa PJ, Etchells TA, Boracchi P, Biganzoli E, Ellis IO (2010). A methodology to identify consensus classes from clustering algorithms applied to immunohistochemical data from breast cancer patients. Comput Biol Med.

[13] Tadrous PJ (2010). On the concept of objectivity in digital image analysis in pathology. Pathology.

[14] Heindl A, Nawaz S, Yuan Y (2015). Mapping spatial heterogeneity in the tumor microenvironment: a new era for digital pathology. Lab Invest.

[15] Carvajal-Hausdorf DE, Schalper KA, Neumeister VM, Rimm DL (2015). Quantitative measurement of cancer tissue biomarkers in the lab and in the clinic. Lab Invest.

[16] Laurinavicius A, Laurinaviciene A, Ostapenko V, Dasevicius D, Jarmalaite S, Lazutka J (2012). Immunohistochemistry profiles of breast ductal carcinoma: factor analysis of digital image analysis data. Diagn Pathol.

[17] Budczies J, Klauschen F, Sinn BV, Gyorffy B, Schmitt WD, Darb-Esfahani S, Denkert C (2012). Cutoff Finder: a comprehensive and straightforward Web application enabling rapid biomarker cutoff optimization. PLoS One.

[18] Han HJ, Russo J, Kohwi Y, Kohwi-Shigematsu T (2008). SATB1 reprogrammes gene expression to promote breast tumour growth and metastasis. Nature.

[19] Patani N, Jiang W, Mansel R, Newbold R, Mokbel K (2009). The mRNA expression of SATB1 and SATB2 in human breast cancer. Cancer Cell Int.

[20] Yamayoshi A, Yasuhara M, Galande S, Kobori A, Murakami A (2011). Decoy-DNA against special AT-rich sequence binding protein 1 inhibits the growth and invasive ability of human breast cancer. Oligonucleotides.

[21] Hanker LC, Karn T, Mavrova-Risteska L, Ruckhaberle E, Gaetje R, Holtrich U, Kaufmann M, Rody A, Wiegratz I (2011). SATB1 gene expression and breast cancer prognosis. Breast.

[22] Kohwi-Shigematsu T, Han HJ, Russo J, Kohwi Y (2010). Re: The role of SATB1 in breast cancer pathogenesis. J Natl Cancer Inst.

[23] Iorns E, Hnatyszyn HJ, Seo P, Clarke J, Ward T, Lippman M (2010). The role of SATB1 in breast cancer pathogenesis. J Natl Cancer Inst.

[24] Heubner M, Kimmig R, Aktas B, Siffert W, Frey UH (2014). The haplotype of three polymorphisms in the promoter region impacts survival in breast cancer patients. Oncology letters.

[25] Kobierzycki C, Wojnar A, Dziegiel P (2013). Expression of SATB1 protein in the ductal breast carcinoma tissue microarrays - preliminary study. Folia Histochem Cytobiol.

[26] Liu X, Zheng Y, Qiao C, Qv F, Wang J, Ding B, Sun Y, Wang Y (2015). Expression of SATB1 and HER2 in breast cancer and the correlations with clinicopathologic characteristics. Diagn Pathol.

[27] Brocato J, Costa M (2015). SATB1 and 2 in colorectal cancer. Carcinogenesis.

[28] Selinger CI, Cooper WA, Al-Sohaily S, Mladenova DN, Pangon L, Kennedy CW, McCaughan BC, Stirzaker C, Kohonen-Corish MR (2011). Loss of Special AT-Rich Binding Protein 1 Expression is a Marker of Poor Survival in Lung Cancer. J Thorac Oncol.

[29] Elebro J, Heby M, Gaber A, Nodin B, Jonsson L, Fristedt R, Uhlen M, Jirstrom K, Eberhard J (2014). Prognostic and treatment predictive significance of SATB1 and SATB2 expression in pancreatic and periampullary adenocarcinoma. Journal of translational medicine.

[30] Keith B, Johnson RS, Simon MC (2011). HIF1alpha and HIF2alpha: sibling rivalry in hypoxic tumour growth and progression. Nature reviews Cancer.

[31] Schindl M, Schoppmann SF, Samonigg H, Hausmaninger H, Kwasny W, Gnant M, Jakesz R, Kubista E, Birner P, Oberhuber G (2002). Overexpression of hypoxia-inducible factor 1alpha is associated with an unfavorable prognosis in lymph node-positive breast cancer. Clin Cancer Res.

[32] Bos R, van der Groep P, Greijer AE, Shvarts A, Meijer S, Pinedo HM, Semenza GL, van Diest PJ, van der Wall E (2003). Levels of hypoxia-inducible factor-1alpha independently predict prognosis in patients with lymph node negative breast carcinoma. Cancer.

[33] Yamamoto Y, Ibusuki M, Okumura Y, Kawasoe T, Kai K, Iyama K, Iwase H (2008). Hypoxia-inducible factor 1alpha is closely linked to an aggressive phenotype in breast cancer. Breast Cancer Res Treat.

[34] Dales JP, Beaufils N, Silvy M, Picard C, Pauly V, Pradel V, Formisano-Treziny C, Bonnier P, Giusiano S, Charpin C, Gabert J (2010). Hypoxia inducible factor 1alpha gene (HIF-1alpha) splice variants: potential prognostic biomarkers in breast cancer. BMC medicine.

[35] Vleugel MM, Greijer AE, Shvarts A, van der Groep P, van Berkel M, Aarbodem Y, van Tinteren H, Harris AL, van Diest PJ, van der Wall E (2005). Differential prognostic impact of hypoxia induced and diffuse HIF-1alpha expression in invasive breast cancer. J Clin Pathol.

[36] Rundqvist H, Johnson RS (2013). Tumour oxygenation: implications for breast cancer prognosis. Journal of internal medicine.

[37] Wockel A, Wolters R, Wiegel T, Novopashenny I, Janni W, Kreienberg R, Wischnewsky M, Schwentner L (2014). The impact of adjuvant radiotherapy on the survival of primary breast cancer patients: a retrospective multicenter cohort study of 8935 subjects. Ann Oncol.

[38] Konig J, van Ewijk R, Kuhr K, Schmidberger H, Wockel A, Kreienberg R, Blettner M (2015). Radiotherapy effects on early breast cancer survival in observational and randomized studies: a systematic analysis of advantages, disadvantages and differences between the two study types. Breast Cancer.

